# An Outbreak of Rift Valley Fever in Northeastern Kenya, 1997-98

**DOI:** 10.3201/eid0802.010023

**Published:** 2002-02

**Authors:** Christopher W. Woods, Adam M. Karpati, Thomas Grein, Noel McCarthy, Peter Gaturuku, Eric Muchiri, Lee Dunster, Alden Henderson, Ali S. Khan, Robert Swanepoel, Isabelle Bonmarin, Louise Martin, Philip Mann, Bonnie L. Smoak, Michael Ryan, Thomas G. Ksiazek, Ray R. Arthur, Andre Ndikuyeze, Naphtali N. Agata, Clarence J Peters, the World Health Organization Hemorrhagic Fever Task Force

**Affiliations:** * Centers for Disease Control and Prevention, Atlanta, Georgia, USA; † European Program for Intervention Epidemiology Training (EPIET), European Union, Saint-Maurice, France; EPICENTRE, Paris, France; § Kenya Ministry of Health, Nairobi, Kenya; ¶ Kenyan Medical Research Institute, Nairobi, Kenya; # National Institutes of Virology (NIV), Sandringham, South Africa; ** World Health Organization, African Regional Office, Harare, Zimbabwe; †† Médècins Sans Frontieres, Paris, France; ‡‡ United States Army Medical Research Unit, Kenya; §§ International Federation of the Red Cross and Red Crescent, Geneva, Switzerland; ¶¶ Médècins du Monde, Paris, France; ## Africa Medical Research Foundation, Nairobi, Kenya

**Keywords:** Rift Valley fever, Kenya, zoonosis, hemorrhagic fever

## Abstract

In December 1997, 170 hemorrhagic fever-associated deaths were reported in Carissa District, Kenya. Laboratory testing identified evidence of acute *Rift Valley fever virus* (RVFV). Of the 171 persons enrolled in a cross-sectional study, 31(18%) were anti-RVFV immunoglobulin (Ig) M positive. An age-adjusted IgM antibody prevalence of 14% was estimated for the district. We estimate approximately 27,500 infections occurred in Garissa District, making this the largest recorded outbreak of RVFV in East Africa. In multivariate analysis, contact with sheep body fluids and sheltering livestock in one’s home were significantly associated with infection. Direct contact with animals, particularly contact with sheep body fluids, was the most important modifiable risk factor for RVFV infection. Public education during epizootics may reduce human illness and deaths associated with future outbreaks.

*Rift Valley fever virus* (RVFV) is a zoonosis that can cause epizootics and associated human epidemics in Africa (*1*). RVFV is a member of the family Bunyaviridae, genus *Phlebovirus*. Epizootics of RVFV occur periodically after heavy rains that flood natural depressions in the grasslands of sub-Saharan Africa (*2*). Flooding allows for the hatching of the primary vector and reservoir, multiple species of mosquitoes known as floodwater *Aedes*, which feed on nearby mammals (*3*,*4*). High levels of viremia in these animals lead to infection of secondary arthropod vector species and to subsequent infection of other mammals and livestock, in which it causes abortions and death in susceptible animals (*5*–*7*).

Human infection with RVFV was first reported soon after Daubney and colleagues isolated the virus in 1930 (*8*). Extensive human disease outbreaks were not reported until 1951, however, when an estimated 20,000 persons were infected during an epizootic of cattle and sheep in South Africa (*9*). Outbreaks were reported exclusively from sub-Saharan Africa until 1977-78, when 18,000 persons were infected and 598 deaths were reported in Egypt (*10*). Transmission of the virus to humans is thought to occur by arthropod vectors, aerosols of blood or amniotic fluid, or other direct contact with infected animals. RVFV in humans manifests a broad spectrum of disease, from asymptomatic infection to a benign febrile illness, to a severe illness in approximately 1%-3% of cases that can include retinitis, encephalitis, and hemorrhagic fever (*11*,*12*). In addition to the human illness, disability, and suffering, RVFV outbreaks can result in devastating economic losses when livestock in an agricultural society are affected (*13*,*14*).

The normalized difference vegetation index has a linear relationship to rainfall in semiarid regions such as East Africa. Figure 1 depicts the vegetation development (as sensed by orbiting satellites) in late 1997, indicating elevated rainfall and the potential for RVFV outbreaks (*15*). In December 1997, the Kenyan Ministry of Health (MOH) and the World Health Organization (WHO) in Nairobi received reports of unexplained deaths in the North Eastern Province of Kenya and southern Somalia. Clinical features included an acute onset of fever and headache often associated with hemorrhage (hematochezia, hematemesis, and bleeding from other mucosal sites). Farmers and local veterinary health officials also reported high rates of spontaneous abortion and death among domestic livestock. Active surveillance conducted by the WHO, the Kenya MOH, and international relief organizations during December 22 to 28 in 18 villages in Garissa District (population 231,022), North Eastern Province, Kenya, identified 170 human deaths, reportedly from a hemorrhagic fever. No clinical specimens were available from the persons who had died. However, of 36 blood samples obtained from other ill persons in these villages that were tested at the National Institute for Virology and at the Centers for Disease Control and Prevention (CDC), 17 (47%) had evidence of acute infection with RVFV by detection of IgM antibodies, virus isolation, or reverse-transcriptase-polymerase chain reaction (RT-PCR) for viral nucleic acid.

**Figure 1:**
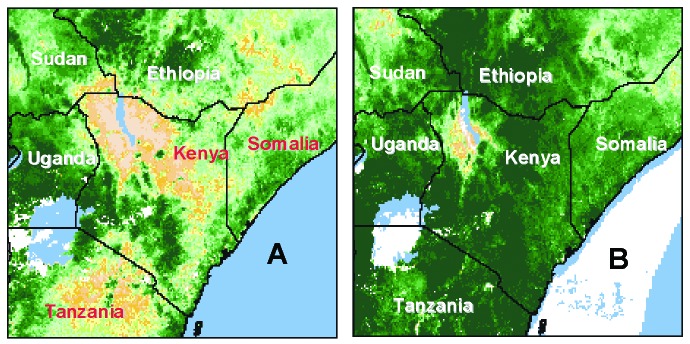
Images from advanced, very high resolution radiometer instrument on a National Oceanic and Atmospheric Administration satellite comparing normalized difference vegetation index data (as a surrogate for rainfall), from December 1996 (A) and December 1997 (B). Increasing vegetation is depicted from tan to yellow [predominating in part (a)], to light and dark green [predominating in (b)].

After RVFV was identified as the primary cause of this outbreak, an international task force led by the Kenya MOH established surveillance for hemorrhagic fever and other severe manifestations of infection. Because the surveillance system identified only those with severe symptoms, the task force also conducted a cross-sectional study in the human population of the Garissa District to determine the incidence of recent infection with RVFV and to evaluate risk factors for infection.

## Methods

### Site and Population

The investigation was conducted in the Garissa District of the North Eastern Province of Kenya, which borders southern Somalia. In 1998, an estimated 231,022 residents living in Garissa District were distributed among 12 divisions and 84 sublocations (Office of the President, Garissa District Development Plan: 1997-2001, unpub. data). In addition to the Kenyan residents, 120,000 Somali refugees lived in the district in 1998. Garissa has an arid climate with a predominantly flat and grassy landscape. Precipitation in the district averages 250 mm to 500 mm annually, varies considerably from year to year, and occurs in a bimodal pattern with two peaks, in March through May and October through December. Although a number of settled towns are dispersed throughout the region, the rural population is principally composed of nomadic herdsmen.

Two events hampered surveillance. By January 1998, floodwater had damaged or covered most of the already poor roadways in the Garissa District, making them impassable. Investigators required air transportation to perform surveillance. Additionally, a nationwide nurses’ strike in December and January greatly decreased the medical personnel in hospitals and clinics, which contributed to the difficulties in providing patient care and collecting data.

### Hemorrhagic Fever Surveillance

#### Case Definition

A probable case was defined as someone presenting with fever and bleeding from their gums, nose, eyes, rectum, lungs, or gastrointestinal tract, between October 1997 (the onset of flooding) and February 8, 1998. If specimens were available, diagnostic laboratory tests for RVFV were performed.

#### Case Ascertainment

In Garissa, active case-finding for hemorrhagic fever was performed by members of local law enforcement, village chiefs, and other government officials. Periodic reports were provided to the district health officers and the task force. We disseminated a case-report form to all district medical officers and international relief agencies involved in medical care in the area. When possible, reports of possible cases were investigated to obtain demographic and clinical information, as well as blood, stool, or tissue specimens. The WHO Hemorrhagic Fever Task Force was conducting similar surveillance throughout Kenya and in southern Somalia and northern Tanzania.

#### Laboratory

Initially, specimens were transported to the Africa Medical Research Foundation in Nairobi, where serum specimens were centrifuged, divided into aliquots, and forwarded to the Viral Research Center/Kenyan Medical Research Institute. Subsequently, a laboratory operated by Médècins du Monde was established in Garissa to process clinical samples. Within 12 hours of collection, serum specimens were stored at 4°C until packed and shipped to National Institute for Virology and CDC for RVFV-specific IgG and IgM antibody enzyme-linked immunosorbant assay (ELISA), virus isolation, RT-PCR and sequencing of the DNA product, or immunohistochemistry using previously described methods (*16*–*19*).

### Cross-Sectional Survey

#### Case Definition

For the cross-sectional survey, a laboratory-confirmed case definition was used. A case of recent infection was defined as presence of RVFV-specific IgM antibodies by ELISA. Persons with RVFV-specific IgG antibodies by ELISA and no IgM antibody were considered to have been infected before this outbreak. In our initial calculations, we considered persons with only anti-RVFV IgG antibodies to be protected against infection. The background prevalence of past RVFV infection in Kenya is not known, but IgG positivity as high as 40% has been shown in populations at high risk after epizootics (*20*). In contrast, a 1983 study for serologic prevalence of hemorrhagic fever viruses in Kenya demonstrated that <1% of persons sampled had anti-RVFV IgG, but a less sensitive immunofluorescent antibody test was used (*21*). This study did not include the North Eastern Province. Additionally, the duration of IgM antibodies is not well documented. Therefore, our analysis included persons with either anti-RVFV IgG or IgM antibodies.

#### Sampling

We selected participants for the cross-sectional survey from the non-refugee population of Garissa District using a modified multistage cluster design (*22*). In Kenya, each province is divided into divisions that are made up of locations and then sublocations. We used sublocations as the units for choosing clusters. Thirty clusters (sublocations) were selected from 12 divisions. The number of clusters per division was weighted to represent population density. Clusters were then chosen randomly. Seven households were chosen in each cluster by systematic random sampling for a total sample size of 210 persons. The investigating team identified one person in each household for recruitment into the study. To reflect the age distribution of the RVFV infection and hemorrhagic fever cases already identified in the outbreak, we included one child between 2 and 9 years old, five persons between 10 and 49 years old, and one person ³50 years in each cluster. Between February 8 and 14, 1998, three field teams interviewed and obtained samples from 202 persons from 29 clusters. Three of the selected cluster sites had been destroyed by the flood and could not be located. Replacement sublocations were sampled for two of the three destroyed clusters. In one cluster, only six persons were sampled. In four clusters, children <10 years of age were not sampled and were replaced by adults.

#### Data Collection

After informed consent was obtained, a blood specimen was collected and each participant was interviewed. We used a standardized questionnaire that included demographic characteristics (age, gender, family size), exposure information (e.g., slaughtering practices, butchering, consuming raw meat and milk), environmental factors (displacement by flood, type of settlement, loss of livestock), and history of illness between the start of the floods and the date of the interview. Exposure to mosquitoes was evaluated through questions about attempts to reduce bites (i.e., mosquito nets, fires, other methods). Otherwise, all persons were assumed to share a similar risk for insect bites. Local health workers fluent in English, Kiswahili, and Somali were trained to administer the questionnaire and were supervised by an epidemiologist. Interviewers recorded information in English.

#### Laboratory

Blood specimens were kept at ambient temperature for <6 hours. Specimens were processed at the Médècins du Monde laboratory as noted above. RVFV-specific IgG ELISA, as well as IgM ELISA, was performed on all blood specimens. At the time of the cross-sectional survey, there were no reports of severe manifestations of illness and we believed that virus transmission was not ongoing. Therefore, virus isolation and PCR were not performed on these specimens.

#### Data Management and Analysis

Data from completed questionnaires were double-entered into databases by using Epi Info version 6.0 (CDC, Atlanta, GA). Univariate and multivariate analyses were performed by using SAS (version 6.12, Cary, NC). Poisson regression was used with a generalized estimating equations algorithm to control for the clustered nature of the data (*23*).

## Results

### Hemorrhagic Fever Surveillance

**Figure 2:**
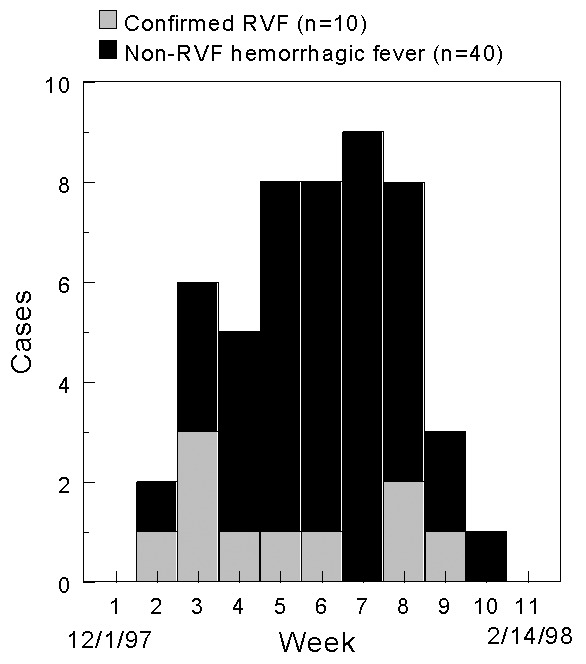
Temporal distribution of hemorrhagic fever cases, by date of onset, Garissa District, Kenya, December 1, 1997 to February 14, 1998. Source: Morbidity and Mortality Weekly Report 1998;47:261-4.

**Figure 3:**
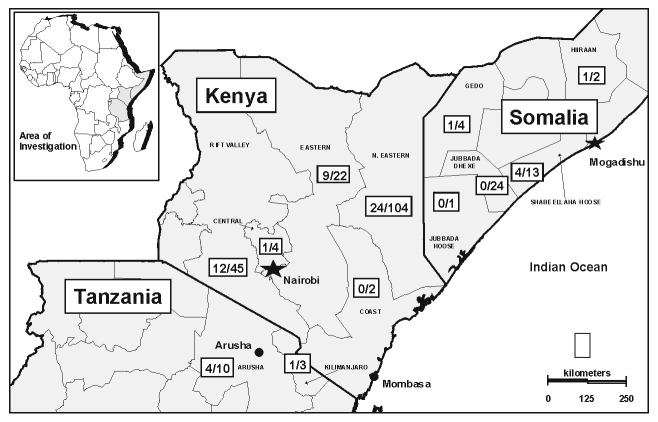
Geographic distribution of Rift Valley fever outbreak, East Africa, 1997-98. (Number of confirmed cases / number of cases with severe febrile illness reported to surveillance system). Source: Morbidity and Mortality Weekly Report 1998;47:261-4.

The hemorrhagic fever surveillance system identified 77 persons with severe febrile illness in Garissa District whose onset of fever was between November 10, 1997, and February 8, 1998. Fifty-three males (57% male; median age 28 years, from 3 to 85 years) met the case definition for hemorrhagic fever (Figure 2). Of these 53 patients, 10 (19%) had evidence of acute RVFV infection (Table 1), and another 10 were positive by the anti-RVFV IgG ELISA. Because few cases were available for follow-up, case-fatality proportions were not calculated. Of the 24 persons whose illness did not meet the case definition for hemorrhagic fever, six (25%) had evidence of acute RVFV infection; one of these six had encephalitis and another had retinitis. The limited surveillance system also confirmed human disease in Tanzania and in Somalia (Figure 3).

**Table 1:** Results of testing for laboratory-confirmed cases of Rift Valley fever, Garissa District, Kenya, 1997–98

Onset date	Collection date	Virus isolation	RT-PCR	IgM ELISA	IgG ELISA
12/9/97	12/23/97	+	+	-	-
12/18/97	12/25/97	+	+	+	-
12/18/97	12/26/97	-	Not done	+	+
12/19/97	12/26/97	+	+	+	+
12/21/97	12/26/97	+	+	+	-
12/22/97	01/22/98	Not done	Not done	+	+
12/30/97	01/23/98	Not done	+	+	+
1/18/98	01/22/98	Not done	-	+	+
1/28/98	02/02/98	Not done	Not done	+	+
2/7/98	02/09/98	Not done	Not done	+	-

RT-PCR=reverse transcription-polymerase chain reaction; IG=immunoglobulin; ELISA=enzyme-linked immunosorbent assay.

### Cross-Sectional Survey

#### Antibody Prevalence

Of the 202 persons enrolled in the cross-sectional study, 31 (15%) were positive only for anti-RVFV IgG (i.e., previously infected). Although persons having only IgG antibody were widely dispersed geographically, the highest prevalence was found in the Hulugho Division (32%) and the Masalani Division (29%). The highest percentage of previous infection by age group was for persons ³65 years of age (Table 2). Characteristics of the sampled population are provided in Table 2.

**Table 2:** Demographic characteristics of persons enrolled in Rift Valley fever cross-sectional survey, Garissa District, Kenya, 1997-98

Characteristic	Total (%) n = 202	Prior^a^ infection (% of total) n=31	Susceptible^b^ n=171	Acute^c^ infection (% of susceptible) n=31
Age group (years)				
<15	40 (20)	2 (5)	38	2 (5)
15 to 65	150 (74)	25 (17)	125	28 (22)
>65	12 (6)	4 (33)	8	1 (13)
Sex				
Male	103 (51)	16 (16)	87	18 (21)
Female	99 (49)	15 (15)	84	13 (15)
Rural habitation	161 (80)	25 (16)	136	30 (22)
Household >4 persons	74 (37)	10 (14)	64	21 (33)

^a^Anti-RVF IgG antibody-positive (no immunoglobulin M [IgM]).

^b^Total screened minus those with prior infection.

^c^Anti-*Rift Valley fever virus* IgM antibody-positive.

Of the 171 susceptible persons in the sample, 31 (18%) were positive for anti-RVFV IgM. The percentage of those with detectable IgM antibody varied with age; this antibody was detected in 5% of children <15 years of age, 23% of persons 15 to 65 years of age, and 13% of persons >65 (Table 2). The age-adjusted prevalence of IgM antibody positivity standardized to the population of Garissa District was 14% (95% CI 11-16). Sixteen clusters, representing all 12 of the administrative divisions in Garissa District, had persons with anti-RVFV IgM antibody, indicating recent viral transmission. Of the 31 persons with serologic evidence of recent infection, 30 (97%) reported having a recent illness, compared with 116 (83%) of the antibody-negative persons (p=0.06); no symptoms distinguished recent RVFV-associated illness from other illness.

Assuming an age-adjusted IgM antibody prevalence of 14% in the susceptible population, we estimate that approximately 27,500 persons were infected with RVFV during this outbreak in Garissa District alone. If the presence of IgG antibody were included in the case definition for recent infection, then the standardized prevalence would increase to 23% (95% CI 20-26) and would represent approximately 53,000 infected residents.

#### Risk Factor Assessment

Certain demographic characteristics (Table 2) were associated with higher rates of infection as determined by IgM ELISA, including rural habitation (p=0.02) and household size of less than four persons (p=0.001). Age <15 years was associated with a lower rate of recent infection (p=0.05). A large number of animal contact activities, including herding, milking, slaughtering, and sheltering animals in the home, were statistically associated with recent RVFV infection (Table 3). The association was greatest with sheep-related activities, especially for those resulting in contact with sheep blood or body fluids.

**Table 3:** Exposures during previous 90 days, Rift Valley fever (RVF) cross-sectional survey, Garissa District, Kenya, 1997-98

	Acute^a^ infection (%) n = 31	No infection (%) n = 140	Relative risk	95% CI
Animal exposures				
Sheltered livestock in home after flood	27 (87)	63 (45)	5.3	2.3-12.6
Killed an animal	20 (64)	47 (34)	2.4	1.3-4.3
Butchered an animal	14 (45)	33 (24)	2.0	1.1-3.6
Skinned an animal	20 (65)	38 (27)	2.4	1.6-3.5
Cooked with meat	20 (65)	48 (34)	2.3	1.1-4.9
Milked animals	25 (80)	59 (42)	3.8	1.9-7.7
Drank raw animal milk	30 (97)	89 (64)	8.6	2.0-36.0
Care of animal during birth	21 (68)	46 (33)	2.6	1.4-4.9
Disposal of aborted fetus	19 (61)	36 (26)	2.8	1.5-5.5
Sheep contact^b^	25 (81)	48 (29)	6.3	2.9-14.0
Goat contact^b^	28 (90)	91 (65)	3.1	1.6-6.4
Cow contact^b^	20 (65)	49 (35)	2.4	1.3-4.5
Camel contact^b^	5 (16)	17 (12)	1.3	0.5-3.8
Non-animal exposures				
Home flooded since November 1997	25 (81)	103 (73)	1.3	0.8-2.1
Ill family member	7 (23)	20 (14)	1.6	0.8-3.1
Contact with a dead human body	6 (21)	10 (7)	2.2	1.0-4.6
Use mosquito nets	19 (61)	102 (73)	0.7	0.3-1.4

^a^Anti-*Rift valley fever virus* immunoglobulin M antibody-positive.

^b^Contact includes herding, cooking, slaughtering or other body fluid contact (except consumption), drinking raw milk.

Many of the animal contact variables were highly associated with infection in univariate analysis, but were also highly colinear. Multivariate analysis of composite variables for species-specific activities that resulted in similar exposures and potential confounders demonstrated a significant association between recent RVFV infection and persons who had contact with sheep blood, amniotic fluid, or milk (not including milk consumption; RR 3.0, 95% CI 1.3-6.7) (Table 4). Sheltering any domestic livestock (mostly sheep and goats) in one=s home during the flood also remained independently and significantly associated with infection in the multivariate analysis (RR 3.5, 95% CI, 1.3-9.1). Although not significant in univariate analysis, being male also was significantly associated with infection (RR 1.6, 95% CI 1.0-2.8). Age <15 years was associated with a reduced risk for infection when controlling for the other risk factors (RR 0.3, 95% CI 0.06-1.0). Drinking raw sheep milk was independently associated with infection, but did not reach statistical significance (RR 1.6, 95% CI 0.9-2.9).

**Table 4:** Multivariable risk factor analysis—cross-sectional survey, Garissa District, Kenya, 1997-98

Exposure	Relative risk	95% CI
Contact with sheep blood or body fluids	3.0	1.3-6.7
Sheltering animals in the home	3.5	1.3-9.1
Male gender	1.6	1.0-2.8
Age <15 years	0.3	0.06-1.0
Drinking raw sheep milk	1.6	0.9-2.9

When the analysis was repeated using detection of either anti-RVFV IgM or IgG as the outcome variable, which effectively doubled the number of cases of RVFV, only contact with sheep blood or body fluids and sheltering animals in the home remained significantly associated with illness in the multivariate model (data not shown).

## Discussion

After heavy rainfall in late 1997, an epidemic of Rift Valley fever among humans accompanied an epizootic among ungulates in East Africa. From the cross-sectional investigation in the Garissa District of Kenya, we estimated 27,500 recent human infections with the virus occurred during this period, making this the largest outbreak of RVFV infection ever recorded in sub-Saharan Africa. In addition to Garissa District in the North Eastern Province, we identified recent human infection associated with hemorrhagic fever or encephalitis in four of Kenya’s six provinces during the 1997-1998 outbreak (CDC, unpub. data). Surveillance also confirmed human disease in Tanzania, and, for the first time, in Somalia. Risk factors for human infection identified by this study included a broad array of activities associated with animal exposures, but most significantly, contact with sheep (particularly contact with sheep blood or other body fluids), male gender, and housing animals indoors with household members. Children <15 years of age were significantly less likely to have had recent RVFV infection.

Persons identified by the surveillance system did not undergo thorough clinical and laboratory investigations, and most persons who reported hemorrhaging were not directly observed by a clinician. Laboratory testing also found evidence of infection with other viral agents (*Dengue* and *Bunyamwera* viruses), malaria, shigella dysentery, and leptospirosis as explanations for some of the persons whose illness met the hemorrhagic fever case definition, but who were negative for RVFV infection (CDC, unpub. data). Additionally, the epidemic appears to have preceded a large outbreak of malaria reported from the same region (*24*). Other possible explanations for the persons with fever and hemorrhage who had no laboratory evidence of RVFV infection include an overly sensitive case definition; improper collection, labeling, handling, and transport of samples; other pathogens or toxins; and complications of malnutrition.

In the cross-sectional study, the case definition for recent infection was based on the detection of IgM antibody. Unfortunately, the kinetics of the IgM response to RVFV are not well described. After natural infection, domestic animals lose a detectable amount of IgM antibody within 6 months of infection (*25*). In a large percentage of humans, experimental inoculation with a killed vaccine results in an early IgM response that wanes and is undetectable by 4 to 6 weeks, but this is not a model for natural infection (*26*). Of the few clinical infections that have been followed closely for serologic conversion, IgM antibody appears around day 5, is absent in 50% by day 45, and is undetectable 4 months later (*12*), whereas IgG appears about day 4 and may persist indefinitely at high titer.

All blood specimens for our cross-sectional study were obtained within 12 weeks of the first reported case of hemorrhagic fever in Garissa District. Therefore, the IgM antibodies probably represent recent infection related to the outbreak. If the IgM antibodies disappear quickly, however, the persons in whom we detected only IgG antibody may actually have been infected recently. If we include those persons in the analysis, the incidence of infection could be as high as 23%, representing an additional 25,000 infected persons in Garissa District alone.

Human suffering from RVFV is compounded by the loss of domestic animals. Livestock owners reported losses of approximately 70% of their animals, with the greatest losses among sheep and goats. Other infections thought to contribute to the high illness among livestock during the flooding included nonspecific pneumonia, pasteurellosis, contagious caprine pleuropneumonia, contagious pustular dermatitis, bluetongue, foot rot, and complications of mange (Field Mission of the Food and Agriculture Organization of the United Nations, unpub. data).

Because direct contact with blood or body fluids from viremic animals is an important risk factor for human infection identified by this study, the high number of domestic animal abortions and deaths may have increased the risk for humans developing illness. Many of the early cases were in persons who had recently been involved in the dissection, slaughter, or care of sick animals (*12*,*27*). A high attack rate has been demonstrated for abattoir workers (*28*), herdsmen (*19*), and veterinary personnel (*9*), all of whom have extensive contact with animal blood or other body fluids in the course of their work. In a retrospective investigation in Senegal IgG based on antibody positivity, men who assisted with animal births or abortions, and women who treated ill animals were found to be at increased risk for infection (*29*). Additionally, the virus has been isolated from raw milk, and ingestion of raw milk has been suggested as a risk factor in previous studies (*30*,*31*).

Although any animal that develops a high level of viremia can pose a certain risk for animal-to-human transmission of virus, we found the greatest association with sheep. In past RVFV epizootics, sheep have been the most susceptible domestic animals (*1*,*9*,*32*). After a 2- to 4-day incubation period, young lambs become listless, and fever and bloody diarrhea occur; the case-fatality of 90% to 100% is attributed to hepatic liquefaction (*33*,*34*). The disease manifests similarly in adult sheep, although the case-fatality rate is much lower (20%-30%). As many as 80% of pregnant ewes abort after infection (*9*). The more severe manifestations in sheep are possibly the result of higher levels of viremia (10^10^ suckling mouse intra-cerebral [SMIC] 50% lethal dose/mL), which can exceed those documented in cows and goats (10^8-10^ SMIC 50% lethal dose/mL) (14). Higher levels of viremia with a high rate of abortion and deaths result in an increased likelihood of human contact with an infectious inoculum. If the animal survives acute illness, the virus can be isolated in lower titers for as long as 3 weeks after illness, making it potentially dangerous to slaughter an animal, even after the epizootic appears to be over (*33*,*35*).

From this study it is not possible to identify which cases were infected by mosquitoes and which through direct contact with animals because we did not gather data on the numbers, species, prevalence of RVFV infection, or biting rates of mosquitos at the time of the outbreak. During February 1998, however, 3,180 mosquitoes were collected from three trapping sites in Garissa District. Of the nine captured species, three have been previously implicated in RVFV transmission to humans (*Anopheles coustani, Mansonia africana, and M. uniformis*). In our multivariate model, we assumed that the high mosquito density led to an equal mosquito exposure rate for the entire population. The association of infection with being male and >15 years indicates that persons who are more likely than other groups to perform high-risk behaviors (i.e., adult men) are more likely to be exposed to the virus. All but one (97%) of the persons with acute RVFV infection, as identified by IgM antibody, had recently exposure to either blood, milk, or abortive materials of a sheep or goat. Most of these people (87%) also sheltered animals in their home.

The probability of recurring outbreaks in East Africa and the potential for spread, by either natural or intentional means, to non-disease endemic areas emphasize the necessity of developing and validating methods to predict, prevent, detect, and treat Rift Valley fever. Remote sensing satellite technology, which can predict rainfall patterns likely to result in disease emergence, has been suggested as a means to monitor RVFV activity (*15*,*36*). Longitudinal studies using satellite data to target areas for animal vaccination, enhance surveillance activities for RVFV in animals and humans, and conduct prospective entomologic studies are in progress. However, until these methods are validated, simple public health interventions may greatly reduce transmission of the virus. Control programs aimed at protecting persons during epizootics should include education about the risks of having contact with infected animal body fluids. Although this report highlights the importance of direct animal contact in the transmission of RVFV to humans, the role of arthropod vectors, particularly in the virus life cycle in epizootics, cannot be discounted. Control of mosquito populations during or after heavy rains should be pursued to prevent animal and human infection.
